# Supplementary material for: Cellulose in Foliage and Changes during Seasonal Leaf Development of Broadleaf and Conifer Species
Source: Plants (Basel). 2022 Sep 15;11(18):2412. doi: 10.3390/plants11182412 (PMC9505942; doi:10.3390/plants11182412)
Supplement: Supplementary file 1 [file plants-11-02412-s001.zip › plants-1865024-supplementary.pdf]

Electronic supplementary material to

Cellulose in foliage and changes during seasonal leaf development of broadleaf and conifer species

Zoltan Kern[1,2,*], Adam Kimak[3,$] István Gábor Hatvani[1,2], Daniela M Llanos Campana[4] and Markus Leuenberger[3]

*Correspondence. zoltan.kern@gmail.com (ZK)

To check the potential impurities in the residual of the single-extracted samples and the expected improvement for the multiple-extracted ones Fourier Transform InfraRed spectroscopy (FTIR) in Attenuated Total Reflexion mode (ATR) was used. Infrared spectroscopy uses IR radiation to measure what fraction of the incident radiation is absorbed at a particular wavelength, which can be used to establish semi-quantitative measures of organic matter composition [1-3]. Fourier transform infrared spectroscopy (FTIR) uses Fourier transform to convert the raw wavelength data collected by a detector into spectra [3]. Attenuated total reflectance (ATR) is a rapid technique which is a useful initial step to characterize organic matter with minimal sample preparation [4]: ATR is based on the phenomenon of total internal reflection [5] and measures the changes which occur in an internally reflected infrared beam which come in contact with the sample through a diamond crystal. When the sample is placed in contact with the ATR crystal, the resulting evanescent wave is attenuated in the regions of the IR spectrum where the sample absorbs energy [4]. For each sample, 16 scans were recorded in the 4000 to 400 cm^{-1} spectral range with a spectral resolution of 4 cm^{-1}. The measurements were carried out using a Bruker Vertex 70 spectrometer (Bruker Corporation, Billerica, MA, USA) controlled by OPUS 7.2 software (Bruker Corporation), at the Institute for Geological and Geochemical Research, Research Centre for Astronomy and Earth Sciences, Budapest. Furthermore, we compared the spectra of the residual of the single-extracted and the multiple-extracted samples to cellulose obtained from wood produced by the same procedure [6].

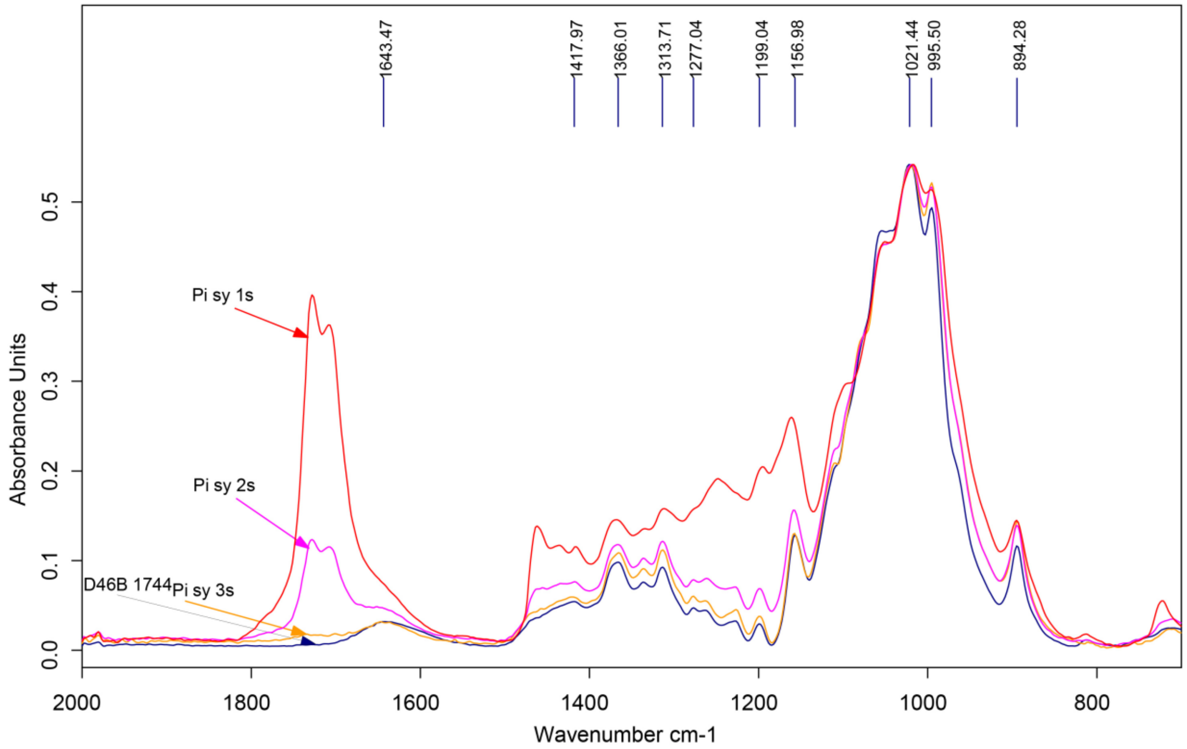

Figure S1. FTIR-ATR spectra of single-extracted (red), double-extracted (purple) and triple-extracted (orange) pine needles and cellulose of trunk wood of *Pinus cembra* [6].

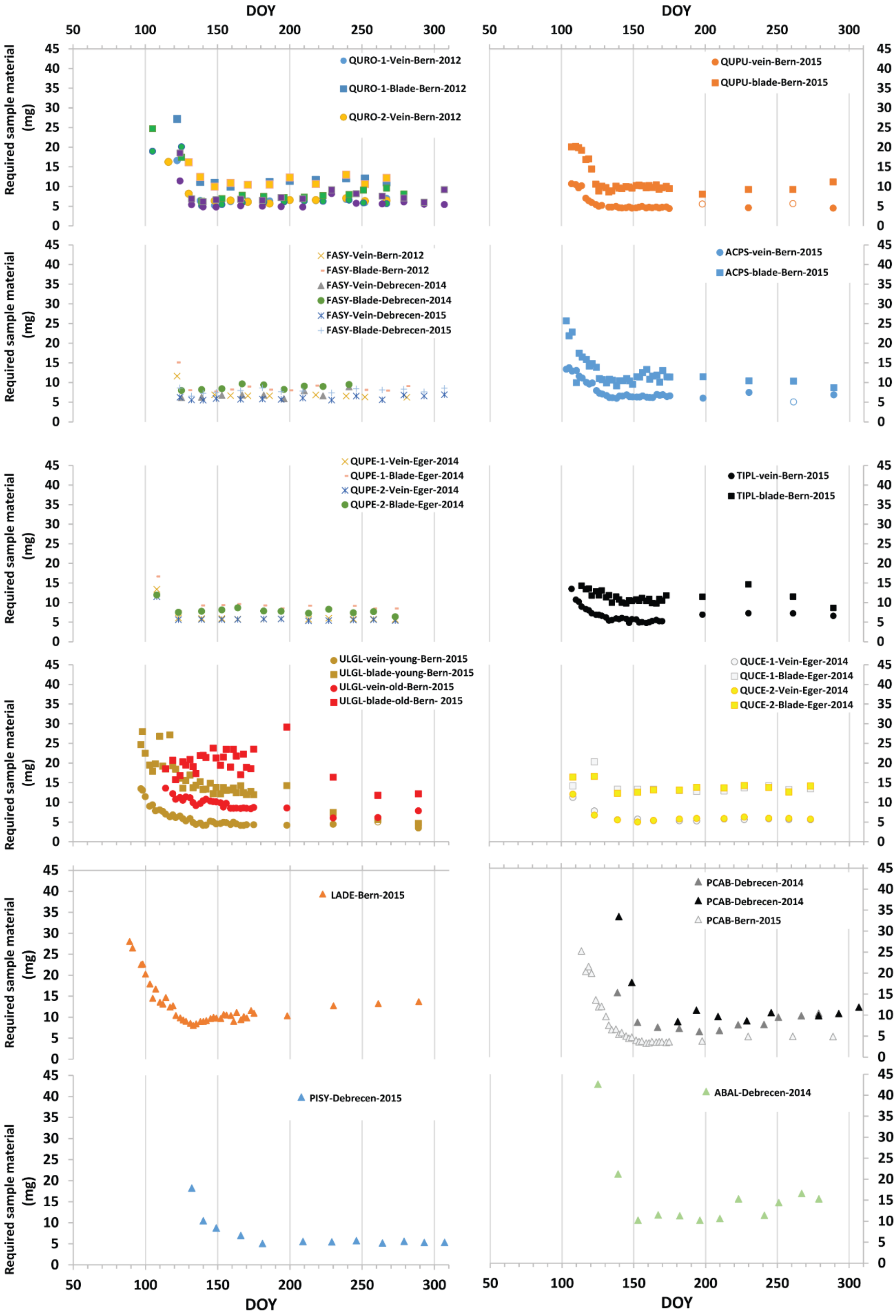

Figure S2. Required sample material (RSM) of foliage in mg vs. day of the year (DOY) of twelve European arboreal species in order to obtain 1 mg alpha-cellulose after extraction by the Jayme-Wise method. Filled squares, circles and triangles denote blades, veins and needle, respectively.

References:

1. Farmer, VC. *The Infrared Spectra of Minerals*. Mineralogical Society of Great Britain and Ireland, London, 1974, 536 pp.
2. Griffiths, P. Fourier transform infrared spectrometry. *Science* **1983,** *222*, 297–302.
3. Thompson, T.J.U.; Gauthier, M.; Islam, M. The application of a new method of Fourier Transform Infrared Spectroscopy to the analysis of burned bone. *J. Archaeol. Sci.* **2009**, *36*, 910–914.
4. Stuart, B. *Infrared Spectroscopy: Fundamentals and Applications.* Wiley, Chichester, 2004, 244 pp.
5. Bruno, T.J., Sampling accessories for infrared spectrometry. *Appl. Spectrosc. Rev.* **1999,** *34*, 91–120.
6. Nagavciuc, V.; Kern, Z.; Perșoiu, A.; Kesjár, D.; Popa, I. Aerial decay influence on the stable oxygen and carbon isotope ratios in tree ring cellulose. *Dendrochronologia* **2018,** *49,* 110-117. doi:10.1016/j.dendro.2018.03.007
